# Methamphetamine enhances caveolar transport of therapeutic agents across the rodent blood-brain barrier

**DOI:** 10.1016/j.xcrm.2021.100497

**Published:** 2022-01-12

**Authors:** Jui-Hsien Chang, Chris Greene, Karen Frudd, Leonardo Araujo dos Santos, Clare Futter, Benjamin J. Nichols, Matthew Campbell, Patric Turowski

**Affiliations:** 1 UCL Institute of Ophthalmology, University College London, London, UK; 2 Smurfit Institute of Genetics, Trinity College Dublin, Dublin 2, Ireland; 3 MRC Laboratory of Molecular Biology, Cambridge, UK

**Keywords:** blood-brain barrier, drug delivery, methamphetamine, glioblastoma, doxorubicin, neurovascular unit, electron microscopy, caveolae, caveolin-1 knockout, ex vivo brain

## Abstract

The blood-brain barrier (BBB) restricts clinically relevant accumulation of many therapeutics in the CNS. Low-dose methamphetamine (METH) induces fluid-phase transcytosis across BBB endothelial cells *in vitro* and could be used to enhance CNS drug delivery. Here, we show that low-dose METH induces significant BBB leakage in rodents *ex vivo* and *in vivo*. Notably, METH leaves tight junctions intact and induces transient leakage via caveolar transport, which is suppressed at 4°C and in caveolin-1 (CAV1) knockout mice. METH enhances brain penetration of both small therapeutic molecules, such as doxorubicin (DOX), and large proteins. Lastly, METH improves the therapeutic efficacy of DOX in a mouse model of glioblastoma, as measured by a 25% increase in median survival time and a significant reduction in satellite lesions. Collectively, our data indicate that caveolar transport at the adult BBB is agonist inducible and that METH can enhance drug delivery to the CNS.

## Highlights

• Low-dose METH induces caveolae in BBB endothelial cells but leaves junctions intact
• These caveolae transport diverse cargo, including chemotherapeutics and antibodies
• Doxorubicin chemotherapy is more efficient in GBM mice when combined with METH

## Graphical abstract

**Figure:**
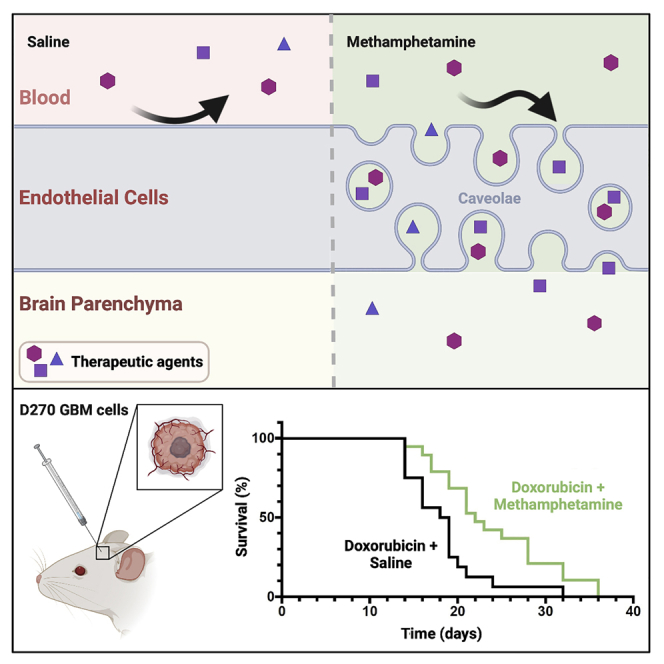

## Teaser

The entry of therapeutics to the brain is strongly limited by the blood-brain barrier. Chang et al. show that in the brain vascular endothelium, low-dose methamphetamine induces caveolae, which can transport varied molecules to the brain and may be used to boost the effectiveness of therapeutics for CNS-driven diseases.

## Introduction

The CNS is highly vascularized such that the disproportional metabolic demand associated with neuronal activity is fully met.^1^ While meeting the metabolic demands of the CNS, its vasculature is selectively and dynamically impermeable to protect the delicate ionic neural environment, a feature referred to as the blood-brain barrier (BBB). The importance of the BBB is illustrated by its dysfunction in a wide variety of CNS diseases.^2^

The BBB is embedded within the neurovascular unit (NVU), comprising vascular endothelial cells, pericytes, astrocytes, and neurons, all of which cooperate tightly to establish and regulate the BBB, both during development and its postnatal maintenance.^1^ The physical barrier of the mammalian BBB is provided mainly by the vascular endothelial cells. BBB endothelial cells have virtually impermeable tight junctions, sealing the paracellular contacts, and a nearly complete absence of fenestrae and fluid-phase endocytosis.^3^ Thus, large and hydrophilic molecules cannot cross the BBB. To enable the entry of nutrients to the CNS, BBB endothelial cells express an array of substrate-specific proteins, which either feed into substrate-specific vesicular transport systems or form highly specific channels or membrane transporters.^4^ Small hydrophobic molecules, which may penetrate the NVU, are mostly eliminated by molecular efflux pumps of the ATP-binding cassette transporter family of proteins.^5^ Consequently, the BBB constitutes a major impediment for the delivery of therapeutics to the CNS, and most drugs do not accumulate at therapeutically required levels in the brain.^6^^,^^7^

The delivery of drugs to the brain has traditionally been investigated in the context of improving chemotherapy-based outcomes for brain tumors, in particular glioblastoma multiforme (GBM). Here, the acuteness of the disease allows for a window to establish a relatively close connection between drug transport and therapeutic effect. However, major research and development efforts have also focused on producing efficient transportation of biologicals to treat neurodegenerative diseases, such as Alzheimer’s or Parkinson’s disease.^8^

A wide variety of strategies have been explored to enhance drug transport to the brain.^7^ Many seek to open the paracellular space between endothelial cells, thus creating a direct passageway between blood and brain parenchyma and enabling enhanced penetration of blood-borne molecules. This can be achieved by osmotically shrinking the endothelial cells,^9^ by focally treating the BBB with a combination of microbubbles and ultrasound,^10^ by stimulation of the BBB with leakage-inducing factors (e.g., bradykinin),^7^ and by interfering with endothelial tight junctions or inducing their targeted downregulation.^11^ Undoubtedly, creating a direct connection between the brain and the circulation bears significant risks, which are well documented and discussed elsewhere.^7^ Therefore, any opening of the BBB to blood constituents needs to be temporally well controlled to avoid significant intoxication of the brain parenchyma with ions and harmful biomolecules. Alternative BBB drug-delivery strategies do not create a direct connection between blood and the brain. These include taking advantage of existing receptor-mediated transcytosis (e.g., of the Fe-transferrin or insulin receptors) to piggyback antibodies, nanocarriers, or engineered viruses to the brain. Importantly, while these strategies leave the BBB physically intact, they require specific adaptation of the drug to a transport system that they are targeted toward.^12^ Another important, mainly auxiliary, strategy aims to reduce or block the elimination of the drugs by ATP-binding cassette transporters.^7^

Circulating methamphetamine (METH) leads to BBB breakdown in rodents.^13^^,^^14^ Based on this feature, METH has been proposed for use to enhance drug transport to the diseased brain.^15^ METH induces BBB breakdown in an endothelial cell-autonomous or -non-autonomous fashion, each governed by a distinct mechanism of BBB opening. At concentrations >10 μM, METH leads to often chronic, endothelial junction breakdown with slow onset.^16^ In contrast, METH at concentrations in the low micromolar range leaves endothelial junctions intact and instead rapidly induces fluid-phase transcytosis in cultured BBB endothelial cells.^17^

Since METH at low concentrations does not induce paracellular opening, we sought to investigate this mechanism further and corroborate its relevance at an intact NVU. We used perfused rodent brains *ex vivo* to show that fluid-phase transcytosis occurred in the intact brain in response to METH and that this involved transport-competent caveolae. METH-induced BBB dysfunction was also corroborated *in vivo*. Lastly, we showed that the efficacy of treating human GBM-bearing mice was enhanced by co-treatment with METH.

## Results and discussion

We developed an *ex vivo*, dual carotid perfused model of the intact rat brain to study BBB leakage in a highly controlled fashion (Figure S1A). When rat heads were perfused via both common carotid arteries at equal pressure, perfusate constituents were durably kept within the side of the head to which they were applied, indicating that under these experimental conditions, significant mixing did not occur at the Circle of Willis (Figure S1B). Thus, this model allowed the study of an experimental condition and its control within the same brain. Preparation of rodent heads and the perfusion protocol was identical to that used to study a fully functional blood-retinal barrier in explants,^18^^,^^19^ suggesting that this protocol left the BBB intact. When heads were perfused with Evans Blue-albumin (EB-Alb), this dye remained restricted to the vasculature of most areas of the brain, including the cortex, hippocampus, and thalamus, and did not leak for at least 1 h (Figure S1C), indicating that BBB properties were preserved.

**Figure 1:**
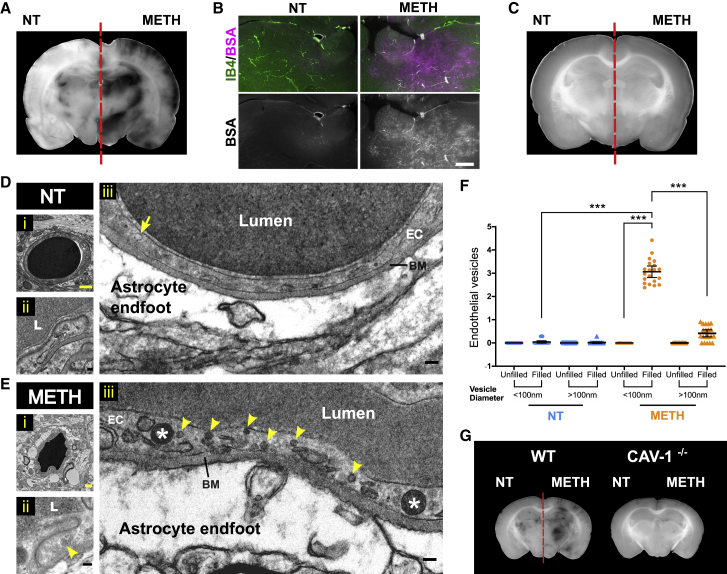
METH induces BBB transport via endothelial caveolae *ex vivo* (A and B) Rat brains were perfused *ex vivo* through both carotid arteries with EB-Alb, as illustrated in Figure S1. METH (1 μM) was included in the perfusate in the right carotid artery. NT, no METH treatment. After 60 min, EB-Alb was flushed from the vasculature, and brains were perfused, fixed, sectioned, and analyzed. Shown in (A) is a bright-field image of a coronal section (representative of n = 5 independent experiments), with the full sectioning profile provided in Figure S2A. Note large parenchymal areas with accumulated EB in the METH but not the NT side of the brain (except in the lateral ventricular areas). Shown in (B) are corresponding fields from the upper medial thalamus, imaged by fluorescent microscopy, with, in the merged images, EB-Alb in magenta and the vasculature (counterstained with isolectin B4 [IB4]) in green. Scale bars, 500 μm. (C) As in (A), except that the head was precooled to 4°C and perfused with ice-cold solutions. Three independent experiments were carried out, and the full sectioning profile is shown in Figure S2E. (D–F) As in (A), but using HRP as a leakage tracer. HRP was not flushed from the vasculature before fixation. Subsequently, corresponding areas in brain sections were analyzed by DAB-EM. Shown in (D) and (E) are vertical sections through representative HRP microvessels in the thalamus and cortex from NT (D) or METH-treated sides (E). Overviews of the vessels are shown in (i), for which magnified intact endothelial paracellular junction areas are shown in (ii) (L = lumen), and ∼3-μm vascular sections in (iii). Arrow, empty intraendothelial vesicle <100 nm; arrowheads, HRP-filled intraendothelial vesicles <100 nm; stars, HRP-filled intraendothelial vesicles >100 nm; EC, endothelial cell; BM, basement membrane. Note the marked enlargement of astrocyte endfeet in response to METH treatment (E), indicating severe perivascular edema. A total of 3 *ex vivo* HRP-perfused rat brains were analyzed by DAB-EM. Yellow scale bars, 1 μm; black scale bars, 1 nm. (F) Vesicle count per micron of endothelial plasma membrane length determined from EM images as in (D) and (E). Data are from 3 independent brains and a total of 20 microvessels. Shown are individual data points, means ± SEMs. ∗∗∗p < 0.001 (ANOVA, Bonferroni post hoc test). (G) As in (A), but performed in either WT or CAV1^−/−^ mice. Note the complete absence of METH-induced leakage in CAV1^−/−^ brains. Shown are representative images from 3 independent experiments. Full sectioning profiles are shown in Figures S2G and S2H.

To analyze leakage across the BBB, METH (1 μM) was included with the EB-Alb-containing perfusate in one side of *ex vivo* brains. After 60 min, the entire head vasculature was cleared to remove intravascular dye. Subsequent analysis of fixed brain slices revealed strong and widespread accumulation of EB-Alb, but only in the hemisphere to which METH was administered (Figures 1 A, 1B, S2A, and S2B). The accumulated EB-Alb was located outside the vasculature (Figures S2C and S2D), indicating that leakage and BBB breakdown had occurred. METH-induced leakage was completely suppressed at 4°C (Figures 1C, S2E, and S2F), demonstrating that it was cold sensitive and thus likely to be dependent on vesicular traffic.^20^^,^^21^ In cultured BBB endothelial cells, METH induces permeability via small pinocytic vesicles with diameters of 70–100 nm, reminiscent of caveolae.^17^ Double carotid perfused brains were treated with or without METH and horseradish peroxidase (HRP) as leakage tracer and brain microvessels analyzed by diaminobenzidine (DAB) electron microscopy (EM) (Figures 1D and 1E). In METH-treated hemispheres, the immediate environment of microvessels displayed perivascular edema, with notable astrocyte endfoot swelling. Accordingly, vessels appeared compressed, with a less rounded appearance and reduced diameter. Paracellular junctions between endothelial cells were ultrastructurally identical to those found in the contralateral control side and did not display any accumulation of DAB, indicating that tight junctions were left intact. However, microvascular endothelial cells in METH-treated hemispheres contained a large number of DAB^+^ vesicles <100 nm diameter, which were uncoated and thus resembled caveolae. Quantification revealed that endothelial accumulation of these vesicles was highly specific to the METH-treated side (Figure 1F). In the METH-treated side, the number of DAB^+^ endothelial vesicles <100 nm was significantly induced. The number of vesicles >100 nm was also significantly increased, but much less strongly. Next, we sought direct proof of caveolae being responsible for BBB tracer leakage in response to METH in our *ex vivo* brain model. For this, the double carotid perfusion model was adapted for use in mice. In wild-type mice, METH induced leakage, which was similar to that seen in rats (Figures 1G, S2G, and S2H). In contrast, in caveolin-1^−/−^ (CAV1^−/−^) mice, which lack caveolae,^22^ there was a complete absence of METH-induced leakage. Thus, we concluded that, as in cultured BBB endothelial cells, METH induced leakage at the intact NVU via transport competent caveolae. This was in agreement with other recent studies, e.g., demonstrating that the initial phase of BBB leakage during experimental stroke is mediated by caveolae.^23^ Furthermore, the lack of pericytes or the pericyte-induced endothelial lipid transporter major facilitator superfamily domain containing 2a (MFSD2A) leads to the upregulation of caveolae at the BBB and leakage.^24^ Lastly, transcellular lymphocyte migration across the BBB also requires caveolar transport,^25^ as does the CNS entry of encephalitic alphaviruses.^26^

We next explored how these *ex vivo* observations translated to the BBB *in vivo*. Previous studies suggest that caveolae-mediated BBB opening is only seen following exposure of BBB endothelial cells to METH in the low micromolar range. We carried out a basic pharmacokinetic study in mice to find conditions that led to low micromolar METH concentration in plasma within 1 h of application. Following a 7.5-mg/kg intraperitoneal (i.p.) bolus injection, plasma METH concentration was ∼1.8-fold higher at 30 min than at 60 min (Table S1), following similar kinetics as reported for METH administered i.p. at 30 mg/kg or intravenously (i.v.) at 10 mg/kg.^14^^,^^27^ Collectively, i.p. injection of METH at 7.5, 2.5, and 0.75 mg/kg led to 1-h plasma concentrations of ∼3, 0.8, and 0.2 μM, respectively. Overall, we concluded that 2.5 mg/kg led to plasma concentrations of ∼1–2 μM within 30 min of injection, and therefore these conditions were used for all subsequent *in vivo* experiments.

**Figure 2:**
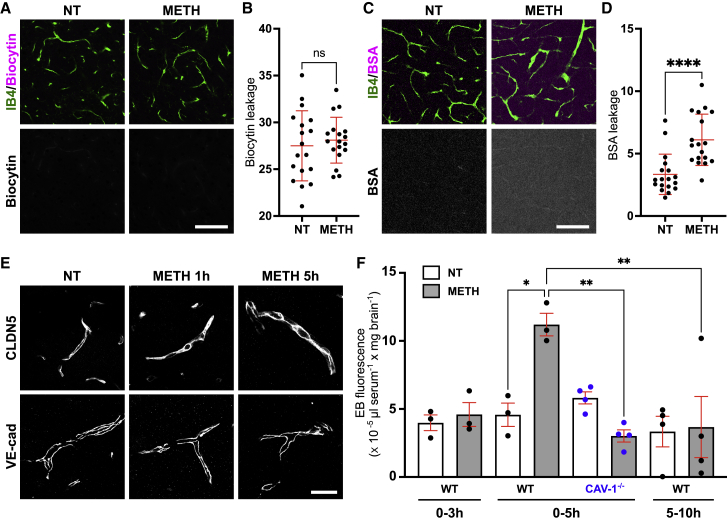
METH induces BBB transport via endothelial caveolae *in vivo* (A–D) C57BL/6 mice were injected i.v. with Alexa Fluor 594 biocytin (A and B) or FITC-BSA (C and D) followed by i.p. injection of METH (2.5 mg/kg) or saline (NT). After 5 h, animals were sacrificed and perfused with 4% paraformaldehyde (PFA). Fixed sections were then counterstained using IB4 and imaged for the presence of perivascular and parenchymal tracers. Shown are representative images from n = 3 independent experiments. Tracer fluorescence was then quantified in 6 brain areas (see Figure S3A) using projections of optical sections spanning 25 μm and normalized to vascular density (B and D). Scale bars, 100 μm. (E) C57BL/6 mice were injected i.p. with METH (2.5 mg/kg) or saline (NT). After 1 or 5 h, brains were removed, frozen, sectioned, and stained for CLDN5 or VE-cadherin. Shown are maximal projections of confocal sections spanning 27 μm (representative of n = 3 independent experiments). Scale bar, 20 μm. (F) WT or Cav1^−/−^ mice were injected i.v. with EB followed by i.p. injection of METH (2.5 mg/kg) or saline (NT) at time 0 (0–3 and 0–5 h) or 5 h (5–10 h). Animals were sacrificed and perfused at 3, 5, or 10 h, the brains isolated and digested and the EB extracted and quantified. Shown is the brain-to-serum ratio of EB fluorescence per brain weight ± SEM and the individual data points from each animal. ns, non-significant, ∗p < 0.05; ∗∗∗p < 0.001 (ANOVA, Bonferroni post hoc test).

Leakage of intravenous biocytin, a tracer with preference for paracellular transit,^23^ was unchanged over a 5-h period in animals treated with 2.5 mg/kg METH (Figures 2A and 2B). In contrast, leakage of fluorescein isothiocyanate (FITC)-BSA, preferentially transported transcellularly,^23^ was significantly enhanced (Figures 2C and 2D). Rates of METH-induced FITC-BSA accumulation varied across brain regions, being lowest in the hippocampus (∼1.8-fold increase) and highest in the striatum (∼4.2-fold) (Figure S3A). CLDN5 and vascular endothelial (VE)-cadherin staining of microvessels was unchanged throughout METH-treated mouse brains (Figures 2E and S3B). This indicated that, as seen *ex vivo*, low METH *in vivo* induced vesicular transport but left paracellular junctions intact. For a more quantitative analysis of METH-induced BSA transport, we used i.v.-injected EB as a tracer. Accumulation of EB in the brain of mice treated simultaneously i.p. with 2.5 mg/kg METH started to be detectable after 3 h and was significantly increased by >2.5-fold after 5 h (Figure 2F). No significant changes in EB brain accumulation were measured in saline-injected control animals. Importantly, when EB was injected 5 h after the METH priming, EB no longer accumulated in the brain, indicating that while METH induced significant BBB opening more slowly *in vivo* than *ex vivo*, leakage was transient and restricted to the first 5 h of METH application. Importantly, EB leakage following METH treatment was completely absent in CAV1^−/−^ mice, demonstrating that, in agreement with our results *ex vivo,* METH-induced BBB opening *in vivo* occurred via transport-competent caveolae.

**Figure 3:**
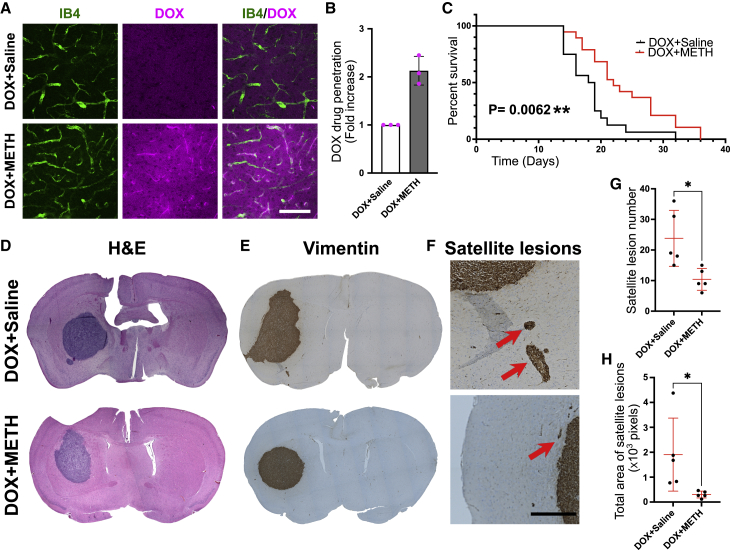
METH enhances DOX-mediated survival in glioma-bearing mice (A and B) Rat brains were perfused *ex vivo* through both carotid arteries with cardioplegic solution containing DOX (10 μg/mL) and METH (1 μM) versus saline in opposing sides as indicated. After 60 min, the vasculature was flushed and brains perfused fixed, sectioned, stained, imaged, and quantified. Shown are representative fields from thalami stained using IB4 (green). DOX was detected by virtue of its autofluorescence (magenta) and accumulated both in the vessels and the parenchyma. Scale bars, 50 μm. (B) Quantification of fluorescent intensity in sections as shown in (A). Shown is DOX fluorescence normalized to vascular (IB4) areas as means ± SEMs from 3 individual brain sections, from n = 3 independent experiments. (C) The right caudate-putamen regions of athymic nude mice were injected with human D270 cells. Subsequently, all of the mice were treated i.p. with 6 mg/kg DOX on days 3, 6, 9, 12, and 15. In addition, mice were randomized and received an additional i.p. injection of saline (n = 16) or METH (2.5 mg/kg) (n = 19). Survival was recorded and is shown; p value was calculated using a Mantel-Cox log-rank test. (D) Representative coronal sections of brains, removed from animals at time of death, as described in (C). Staining using H&E showed similar tumor growth at the time of death. (E–H) Five brains from each group described in (C) were randomly selected, sectioned, and stained using anti-human vimentin antibodies (E). This revealed clear satellite lesions outside the bulk tumors (arrows) (F). Scale bar, 500 μm. The quantification of satellite lesions showed that those in the DOX-METH brains were smaller both in number (G) and size (H). Shown are means ± SEMs and the individual data points from each animal. ∗p < 0.05 (Student’s t test).

We next explored the possibility that METH could enhance BBB transport of therapeutic molecules with different physical and chemical properties. Doxorubicin (DOX), a small (544 g/mol) chemotherapeutic anthracycline with generally poor BBB permeability,^28^ was applied to rat heads via the dual carotid method in the presence and absence of 1 μM METH. After 60 min, brains were isolated and examined for the presence of DOX (by virtue of its autofluorescence). In the absence of METH, DOX autofluorescence was virtually absent from the brain parenchyma (Figures 3A and 3B). In contrast, in the presence of METH, strong DOX autofluorescence was detected on capillaries and throughout the entire brain. Such METH-induced enhancement of DOX to the *ex vivo* brain was not observed in similar experiments carried out in CAV1^−/−^ mice (Figure S4A). Notably, brain accumulation of aflibercept (AFL), used as a paradigmatic large therapeutic protein, was also strongly and significantly enhanced in METH-treated rats *ex vivo* (Figures S4B and S4C). Collectively, these results showed that tracer molecules such as BSA and HRP, but also therapeutics such as DOX and AFL, were excluded from the brain by a functional BBB and that METH enhanced their transport to the brain, suggesting that the BBB delivery of a wide variety of circulating molecules could be enhanced by METH.

Lastly, we studied whether METH enhanced the therapeutic efficacy of DOX in a mouse model of GBM. Brains of BALB/c nude mice were injected with human D270 cells to induce robust formation of tumors bearing many features of human GBM, including the formation of a bulk lesion with a necrotic core, but also substantial invasive satellite growth outside the bulk tumor.^29^ Sixteen days after orthotopical transfer, D270 grew to ∼4- to 5-mm diameter resectable tumors, which contained highly permeable vasculature and a necrotic core (Figures S5A and S5B). Next, cohorts of D270-inoculated BALB/c mice were treated with either DOX (6 mg/kg) or DOX and METH (2.5 mg/kg) on days 3, 6, 9, 12, and 15 after tumor cell inoculation. A significant difference in the survival of the mice undergoing the two different treatment regimens was observed (Figure 3C). Median survival in the DOX-only group (n = 16) was 17.5 (±1.1) days and in line with previous reports for this model.^29^ In contrast, median survival time in the DOX + METH group (n = 19) was 22 (±1.5) days—in other words, significantly increased by ∼25% (p = 0.0062). Importantly, weight loss in both groups was indistinguishable, indicating that differences in survival times were not due to METH improving general health independently (Figure S5C). This survival improvement was similar to that seen when D270 tumor grafts are targeted with specifically designed chimeric antigen receptor T cells.^29^ Postmortem histological analyses of brain sections showed the presence of very large tumor growths, which were clearly identifiable by H&E staining (Figure 3D). Tumors were similar in size in both the DOX only and DOX + METH groups. Staining using anti-human vimentin antibodies revealed that in both groups, there were lesions outside the main bulk tumor areas (Figures 3E and 3F), as also noted by others using this GBM model.^30^ Quantification of these satellite lesions showed that they were significantly less frequent and smaller in size in brains of the METH-treated group (Figures 3G and 3H), suggesting that the METH + DOX regimen affected the BBB outside the main tumor area. While our analyses did not provide a definitive mechanistic reason for enhanced survival in DOX + METH mice, the reduction in satellite lesions appeared relevant. In grade III and IV GBM patients, the extent of multifocality and satellites is significantly linked to reduced survival.^31^

In conclusion, in this proof-of-principle study, we have demonstrated that METH triggered caveolar transport of proteins and small molecules at the intact neurovascular unit and that this process could be harnessed to enhance brain availability of a circulating drug. Caveolae formation in BBB endothelial cells is generally a sign of a dysfunctional BBB. For instance, reduced pericyte coverage leads to endothelial transcytosis, most likely via caveolae.^32^^,^^33^ At the healthy BBB, caveolae formation is functionally suppressed by the MFSD2A, regulating the lipid composition of the inner leaflet of the plasma membrane.^24^ Here, we show that caveolae can also be rapidly induced at the adult BBB by agonists such as METH.

Therapeutic use of METH can be safe, and its use is US Food and Drug Administration (FDA) approved to treat obesity as well as attention-deficit/hyperactivity disorder (https://www.accessdata.fda.gov/drugsatfda_docs/label/2013/0005378s027lbl.pdf). Our study showed that induction of BBB opening occurred with METH at low concentrations and is thus compatible with relative safe use. Other reported METH treatment regimens in rodents generally use much higher concentrations (≥10 mg/kg), which are demonstrably more neurotoxic and induce severe BBB disruption as a consequence of hyperthermia and neuroinflammation.^13^^,^^16^^,^^34^ Furthermore, induction of transport-competent caveolae at the BBB offers clear advantages over opening paracellular junctions, the mechanism invoked by treatment regimens using higher METH concentrations. It allows transport of blood constituents to the brain parenchyma without creating a direct connection between blood and brain, and thus displays much lower toxicity for neuronal networks and, in particular, their delicate ionic environment. Last, and most important, we showed clear therapeutic benefits for METH use in combination with a chemotherapeutic drug in a mouse xenograft glioma model, as measured by survival times. Residence time of METH in the circulation was very short-lived in mice^14^ (Table S1), as it is in humans,^35^ pointing toward a short window of opportunity during which the BBB is open for blood-borne molecules. Indeed, EB accumulation in mice occurred only in the first 5 h of METH treatment, demonstrating that BBB opening by METH constitutes a temporally well-controlled process, highlighting a highly desirable feature due to potentially reduced brain toxicity. We propose that adjunctive METH treatment could be rapidly developed for versatile clinical use. Our data suggest that METH-induced caveolae will transport molecules of widely varying chemical composition and size, raising the likely possibility that even transport of nucleic acids, nanoparticles, and viral particles to the brain can be enhanced by low-level METH.

## Limitations of the study

METH is a highly addictive substance of abuse that can lead to neurotoxic adverse effects. In this study, METH *in vivo* treatment led to plasma concentrations that at least initially exceeded those associated with therapeutic use^36^ and recommended for safe driving, for example (https://mn.gov/law-library-stat/archive/urlarchive/a080579.pdf). Thus, precise pharmacokinetic studies will need to establish treatment regimens that allow METH dosing without exceeding safe plasma concentrations at all times. Very low METH administration at increased frequency is likely to lead to increasing plasma concentrations that could be kept at a plateau of maximally 1 μM.^37^ Alternatively, once the endothelial target of METH responsible for caveolae induction has been identified, molecules that mimic METH in its BBB opening function, but not its excitotoxicity, could be developed for routine clinical use.

Here, we have used orthotopic human xenografts to model GBM in mice. Ideally, further corroboration of our findings in more complex GBM models that are more representative of the current standard therapy of GBM patients^38^ should also incorporate tumor resection and radiotherapy (e.g., as used by Hingtgen et al. ^39^).

Nevertheless, agonist-inducible caveolar transport as an adjuvant strategy will facilitate the preclinical assessment of novel experimental CNS treatments. Furthermore, in conjunction with chemotherapy, it may be of benefit to GBM patients, but also have value for other disease scenarios and treatment modalities, where the BBB continues to be a significant therapeutic impediment, such as rare pediatric cerebral and many neurodegenerative diseases.

## STAR★Methods

### Key resources table

**Table:** 

REAGENT or RESOURCE	SOURCE	IDENTIFIER
**Antibodies**		

Griffonia Simplicifolia Lectin I (GSL I) isolectin B4, Fluorescein	Vector laboratories	Cat#FL-1201; RRID:AB_2314663
Griffonia Simplicifolia Lectin I (GSL I) isolectin B4, Biotinylated	Vector laboratories	Cat#B-1205
Claudin-5 (4C3C2)	Thermo Fisher scientific	Cat# 35-2500; RRID:AB_2533200
goat polyclonal anti-human IgG Fc antibody	Novus Biologicals	Cat#NB7446; RRID:AB_524649
Donkey anti-Goat IgG (H+L) Cross-Adsorbed Secondary Antibody, Alexa Fluor 488	Thermo Fisher Scientific	Cat#A-11055; RRID:AB_2534102
Donkey anti-Mouse IgG (H+L) Highly Cross-Adsorbed Secondary Antibody, Alexa Fluor Plus 555	Thermo Fisher Scientific	Cat#A32773; RRID:AB_2762848
CONFIRM anti-human Vimentin (V9)	Roche	Cat#790-2917; RRID:AB_2687607
Rabbit anti-mouse IgG F(ab')^2^-fragmented/Biotinylated	Dako/Agilent	Cat#E0413
Claudin 5 Polyclonal Antibody	Thermo Fisher Scientific	Cat#34-1600; RRID:AB_2533157
Donkey anti-Rabbit IgG (H+L) Highly Cross-Adsorbed Secondary Antibody, Alexa Fluor 488	Thermo Fisher Scientific	Cat#A-21206; RRID:AB_2535792
Rabbit anti-VE-Cadherin (C-terminal)	This lab, first published and characterized in (Martins et.al, 2013^17^)	N/A

**Chemicals, peptides, and recombinant proteins**		

Evans Blue	Sigma (Merk)	Cat#E2129
Horseradish peroxidase	Sigma (Merk)	Cat#77332
Doxorubicin hydrochloride	Sigma (Merk)	Cat#D1515
Aflibercept (Eylea®)	A gift from Marcus Fruttiger (UCL IoO)	N/A
(+)-Methamphetamine hydrochloride	Sigma (Merk)	Cat#M8750
FITC-BSA	Thermo Fisher Scientific	Cat#A23015
alexa fluor-Biocytin-594	Thermo Fisher Scientific	Cat#A12922

**Critical commercial assays**		

*In Situ* Cell Death Detection Kit, TMR red	Roche	Cat#12 156 792 910
Ventana DAB Map Detection kit	Roche	Cat#760-124

**Experimental models: Cell lines**		

D270 glioblastoma multiforme tumor cells	Lab of Gerald Grant	N/A

**Experimental models: Organisms/strains**		

Wistar rats	Charles River Laboratories Inc. (Oxford, UK)	Cat#619
C57BL6 mice	Charles River Laboratories Inc. (Oxford, UK)	Cat#027
Tie2-GFP CAV1^−/−^ mice	MRC Laboratory of Molecular Biology (Cambridge, UK)	N/A
BALB/c nude mice (CAnN.Cg-Foxn1nu/Crl)	Charles River Laboratories Inc. (Oxford, UK)	Cat#194

**Software and algorithms**		

Prism 8	GraphPad	N/A
ImageJ	Freely available	https://imagej.nih.gov/ij/
Tumor morphology analysis	This paper	https://doi.org/10.5281/zenodo.5779921
Biorender	biorender.com	https://biorender.com/

**Other**		

GIBCO IMPROVED MEM ZINC OPTION 1X	ThermoFisher Scientific	0050009DJ

### Resource availability

#### Lead contact

Further information and requests for resources and reagents should be directed to and will be fulfilled by the lead contact, Patric Turowski (p.turowski@ucl.ac.uk).

#### Materials availability

This study did not generate new unique reagents.

### Experimental model and subject details

#### Animals

Adult female Wistar rats (5-7 weeks, 100-120 g), C57BL/6 mice (6-8 weeks, 15-20 g) and BALB/c nude mice (CAnN.Cg-Foxn1nu/Crl) (8-12 weeks old, 17-20 g) were purchased from Charles River Laboratories Inc. (Oxford, UK).

Tie2-GFP CAV1^−/−^ mice (3-6 months, 20-45 g) were from the MRC Laboratory of Molecular Biology (Cambridge, UK). In CAV1^−/−^ mice, the CAV1 gene was disrupted by a designed targeting vector replacing the first 2 exons by a neomycin resistance cassette.^22^ Mice were backcrossed onto a C57BL/6 background and those used in the study were inbred for at least 2 generations.

All animals were housed in groups of 3-5 in temperature-controlled units with free access to food and water. Littermates were randomly assigned to experimental groups.

All animal procedures were performed in accordance with Animal Welfare Ethical Review Body (AWERB) and Association for Research in Vision and Ophthalmology (ARVO) Statement for the Use of Animals in Ophthalmic and Vision Research guidelines and under either a UK Home Office or a Health Product Regulatory Authority Ireland license, and approval of institutional (UCL Institute of Ophthalmology or Trinity College Dublin) ethics committees.

#### Cells

D270 cells were originally isolated at Duke University.^40^ Frozen aliquots of the cells were thawed and cultured in GIBCO improved MEM zinc option media containing 2.383 g/L HEPES buffer, 5 mg/L insulin, 584 mg/L L-glutamine, 5 μg/L selenium and 2.2 g/L sodium bicarbonate. The cells were maintained at 37°C with 5% CO_2_ and confirmed to be free from mycoplasma contamination.

### Method details

#### *In situ* dual carotid perfusion assay

After CO_2_ asphyxiation, external and internal jugular veins of rats or mice were transected bilaterally. Both common carotid arteries were isolated and cannulated using polyurethane cannulae (3.5 Fr for rat and 1 Fr for mouse). The head vasculature was immediately flushed with heparin (300 U/mL in saline) and then with a cardioplegic solution (10 mM MgCl_2_, 110 mM NaCl, 8 mM KCl, 10 mM HEPES, 1 mM CaCl_2_ and 10 μM isoproterenol, pH 7) in order to reduce thromboembolism, preserve tissue viability, and stabilize the head vasculature.

Subsequently, heads were perfused via bilateral common carotid cannulae with cardioplegic solution containing the leakage tracers, EB-Alb (5 mg/mL Evans Blue, 10% albumin) or HRP (5 mg/mL), and optionally isolectin B4 (IB4), METH, DOX or AFL. METH was given at 1 μM. After incubation of treatments, the remaining intravascular perfusate was flushed out and the vasculature was cleaned by perfusion of cardioplegic solution. Before removal of brain and eyes, perfusion fixation was carried out with 4% paraformaldehyde (PFA) or Karnovsky EM fixatives (3% v/v glutaraldehyde, 1% v/v PFA in 0.08 M sodium cacodylate buffered to pH 7.4 with 0.1 M HCl) for immunohistochemistry or EM studies, respectively. All solutions were administered simultaneously to both hemispheres with an equal delivery pressure throughout the experiments.

#### Immunohistochemistry and histology of *ex vivo* brains

After PFA perfusion fixation, *ex vivo* brains from *in situ* dual perfusion assay were immersed in 4% PFA for 24 h, sectioned into 100 μm and 500 μm slices by Vibratome® 1000 Plus Sectioning System (FEDELCO, S.L.). Retinae were isolated from the eyes and fixed in PFA for 1 h before further processing. 100 μm brain sections and retinae were blocked with 1% FBS, 2X PBS, 3% Triton X-100, 0.5% Tween 20, 0.2% NaN_3_ and stained overnight at 4°C with FITC-conjugated IB4 (FL-1201, Vector laboratories, 1:200) or primary antibody against CLND5 (C43C2, 35-2500, Thermo Fisher scientific, 1:200). AFL was detected using goat polyclonal anti-human IgG Fc antibody (NB7446, Novus Biologicals, 1:200). Primary antibodies were revealed by incubation with matching fluorophore-conjugated secondary antibody (Thermo Fisher Scientific, 1:200) at room temperature for 2 h.

For analysis of tight junctions, brains were flushed with saline to remove blood then snap frozen in isopentane on dry ice. Once frozen, brains were embedded in OCT and kept at −80°C then cut into 10 μm sections on a cryostat. Dried sections were post-fixed in ice cold methanol for 20 min at −20°C. Sections were blocked with 5% NGS, 0.05% Triton X-100 in PBS for 30 min at RT and incubated overnight with primary antibodies against CLDN5 (Thermo Fisher Scientific, 34-1600) and VE-cad.^17^ Corresponding secondary antibodies (Thermo Fisher Scientific) were added for 1 h at RT before final washes and addition of coverslips.

Gross images of 100 and 500 μm brain sections were acquired on a stereo fluorescence microscope (Olympus) equipped with a color camera. For higher resolution imaging, samples were analyzed on an Axioskop or a CLSM 700 confocal laser scanning microscope (Carl Zeiss). Images were processed and staining intensities quantified using ImageJ (NIH).

#### Transmission EM

After perfusion fixation with Karnovsky solution, *ex vivo* brains treated with HRP-containing perfusate (5 mg/mL) were immersed in the same EM fixative for no more than 48 h, sectioned into 100 μm slices by Vibratome®. The 100 μm free-floating sections were incubated in DAB solution (0.075% DAB/0.02% hydrogen peroxide in 0.1 M Tris) for 30 min at room temperature in the dark. After the DAB reaction, specimens were secondarily fixed in 1% Osmium tetroxide/1.5% Potassium ferrocyanide (in H_2_O) for 1.5 h at 4°C in the dark. They were further dehydrated in ethanol, infiltrated with propylene oxide and finally embedded in Epoxy resin after polymerization of Epon overnight at 60°C. Sample blocks were cut into 70 nm ultrasections by Leica EM UC7 Ultramicrotome, mounted onto 300 mesh EM grids and stained with lead citrate. Stained specimen were imaged using a JEM-1400 Transmission EM (JOEL) equipped with a digital camera (Gatan Orius). Images were processed using ImageJ (NIH). For quantification of HRP filled organelles, a total 3 *ex vivo* rat brains were analyzed by EM. Vessels < 10 μm in diameter were imaged from the METH-treated hemispheres and their corresponding control hemispheres. HRP-filled and non-HRP-filled vesicles were counted per EC length (n/μm) and categorized into 4 groups: > 100 nm filled vesicles, < 100 nm filled vesicles, > 100 nm un-filled vesicles and < 100 nm un-filled vesicles.

#### METH dosing *in vivo*

Total 15 C57BL/6 mice, weighing 17.5 g on average, were randomly allocated to 3 dosing groups (3 mice per group): 0.75 mg/kg, 2.5 mg/kg and 7.5 mg/kg, treated for 60 min by intraperitoneal injection; and an additional 7.5 mg/kg group with a shorter treatment time of 30 min. At the times indicated in Table S1, blood was collected by cardiac puncture into heparin-free capillary blood collection tubes and then centrifuged at 1,000 g for 10 min to separate the plasma from whole blood. Plasma samples were immediately snap frozen and kept at −80°C until METH concentrations were measured by LC-MS/MS (Cyprotex, Cheshire, UK).

#### *In vivo* BBB permeability

C57BL/6 and tie2-GFP CAV1^−/−^ mice with an average weight of 20 g received tail vein injections of EB (80 mg/kg), FITC-BSA (100 mg/kg) or Alexa fluor 594 Biocytin (2.5 mg/kg) followed by i.p injection of METH (2.5 mg/kg) or saline. At indicated times, animals were culled by CO_2_ asphyxiation and perfused with saline solution to remove any remaining tracer from the vasculature.

For EB extraction, prior to perfusion, a blood sample was taken via cardiac puncture to control for injection variability, brains were then isolated and homogenized into trypsin solution (2.5 mg/mL). Blood samples were centrifuged at 10,000 g for 15 min to separate the serum which was transferred to fresh tubes and diluted. Brains were digested using trypsin (2.5 g/L) at 37°C overnight until a fully homogeneous solution was obtained. SDS was added to a final concentration of 1% to homogenates and serum to release any further bound EB. Proteins and nucleic acids were precipitated from serum and homogenate samples by addition of trichloracetic acid (TCA, final concentration 30%) vortexed thoroughly to mix and left on ice for 20 min. Finally, samples were centrifuged at full speed for 10 min and supernatants were measured on a fluorescent plate reader (ex: 620 nm, em: 680 nm). Values were normalized against circulating EB and the weight of the extracted brain tissue.

For mice injected with fluorescent tracers, heads were perfused with 4% PFA, then brains removed and emersed in 4% PFA for 24 h. Brains were sectioned on a vibratome as above and the vasculature stained with IB4 before imaging on an Axioskop or a CLSM 700 confocal laser scanning microscope (Carl Zeiss).

#### D270 cell GBM model in athymic nude mice

BALB/c nude mice (CAnN.Cg-Foxn1nu/Crl) were anaesthetised with a mixture of medetomidine/ketamine and placed in a stereotaxic frame. A 1 cm midline incision was made in the scalp and a burr hole was drilled above the frontal cortex (coordinates from bregma: A/p = +0.5 mm; M/L = −2.5 mm; D/V = +3.0 mm). A Hamilton syringe containing 3 μl of D270 cell suspension (1x10^5^ cells) was slowly lowered into the brain and cells were injected at a rate of 0.2 μl/min. The needle was left in place for 5 min to prevent reflux. Animals were sutured and placed in an incubator until they had recovered.

Initial characterization of primary tumors was performed at 16-days post injection of D270 cells, using a dedicated small rodent 7 T MRI system with gadolinium enhancement, located at the Trinity College Institute of Neuroscience (TCIN), Dublin, Ireland (https://www.neuroscience.tcd.ie/technologies/mri.php). Additionally, TUNEL staining was performed on 12 μM cryo-sections using an *in situ* cell death detection kit, (TMR red, Roche) to monitor cell death at the center of the tumor.

For the main study all D270-injected mice received an intraperitoneal injection of DOX (6 mg/kg in saline) on days 3, 6, 9, 12 and 15. Prior to DOX injection, animals were randomized and received an intraperitoneal injection of saline or METH (2.5 mg/kg in saline). Mice were sacrificed when weight loss exceeded 20% total body weight from pre-surgery measurements. Brains were removed and fixed in 4% PFA overnight at 4°C, washed 3 times in PBS and cryoprotected in 10%, 20% and 30% sucrose before being snap-frozen in optimal cutting temperature (OCT) compound.

Cryo-fixed brains in OTC were immersed in 3.7% PFA and embedded in paraffin. Following preparation of the paraffin sections, samples were dewaxed, rehydrated, stained with Harris Haematoxylin (Pioneer Research Chemicals) and 1% Eosin (Pioneer Research Chemicals) using an automated slide stainer. Alternatively, human vimentin immunohistochemical staining of the paraffin sections was performed by using the Ventana Discovery XT instrument (Roche) with Ventana DAB Map detection Kit (760-124, Roche) and pre-treatment of an EDTA equivalent, cell conditioning 1 (CC1, 950-124, Roche). D270 cells were labeled by the anti-human Vimentin antibody (mouse monoclonal, V9, 790-2917, Roche), followed by a mouse secondary antibody (rabbit polyclonal anti-mouse biotin antibody, E0413, Dako, 1:200). H&E and vimentin-stained sections were scanned by EVOS FL auto 2 imaging system (Thermo Fisher Scientific). The described histopathological studies of GBM xenograft mice were conducted at the Division of Neuropathology, UCL Institute of Neurology.

#### Analysis of tumor morphology from histology sections

Images of human vimentin-stained paraffin sections from GBM xenograft mice were further analyzed for differences in satellite tumor cells using a specifically developed computer vision pipeline. Using MATLAB & Simulink (2021a) and its Computer Vision Toolbox, the main tumor was segmented out from the background by the Otsu’s automatic thresholding method and a mask was created (MATLAB Morphological Operations). The mask of main tumor was extended to cover the outmost visible satellite lesion. The satellite lesions on the thresholded images were analyzed by number and size. Artifacts smaller than 2x2 pixels were discarded as noise.

### Quantification and statistical analysis

Statistical analyses were carried out using Prism 8 (GraphPad). Experiment sample sizes were determined empirically; except for experiment in Figure 3 for which power analysis was used to predetermine the sample size (power = 0.8; type I error = 0.05). Data groups were compared by unpaired two-tailed Student’s t, or one-way ANOVA with Bonferroni post hoc tests. Survival data from GBM mice were analyzed by using a Mantel-Cox Log-rank test.

## Data Availability

• All microscopy data reported in this paper will be shared by the lead contact upon request. • All original code has been deposited at Zenodo and is publicly available as of the date of publication. DOI is listed in the Key resources table. • Any additional information required to reanalyze the data reported in this paper is available from the lead contact upon request.

## Supplemental information

Document S1. Figures S1–S5 and Table S1

Document S2. Article plus supplemental information
