# Supplementary material for: Methamphetamine enhances caveolar transport of therapeutic agents across the rodent blood-brain barrier
Source: Cell Rep Med. 2022 Jan 12;3(1):100497. doi: 10.1016/j.xcrm.2021.100497 (PMC8784794; doi:10.1016/j.xcrm.2021.100497)
Supplement: Document S1. Figures S1–S5 and Table S1 [file mmc1.pdf]

Cell Reports Medicine, Volume *3*

Supplemental information

Methamphetamine enhances caveolar transport of therapeutic agents across the rodent blood-brain barrier

Jui-Hsien Chang, Chris Greene, Karen Frudd, Leonardo Araujo dos Santos, Clare Futter, Benjamin J. Nichols, Matthew Campbell, and Patric Turowski

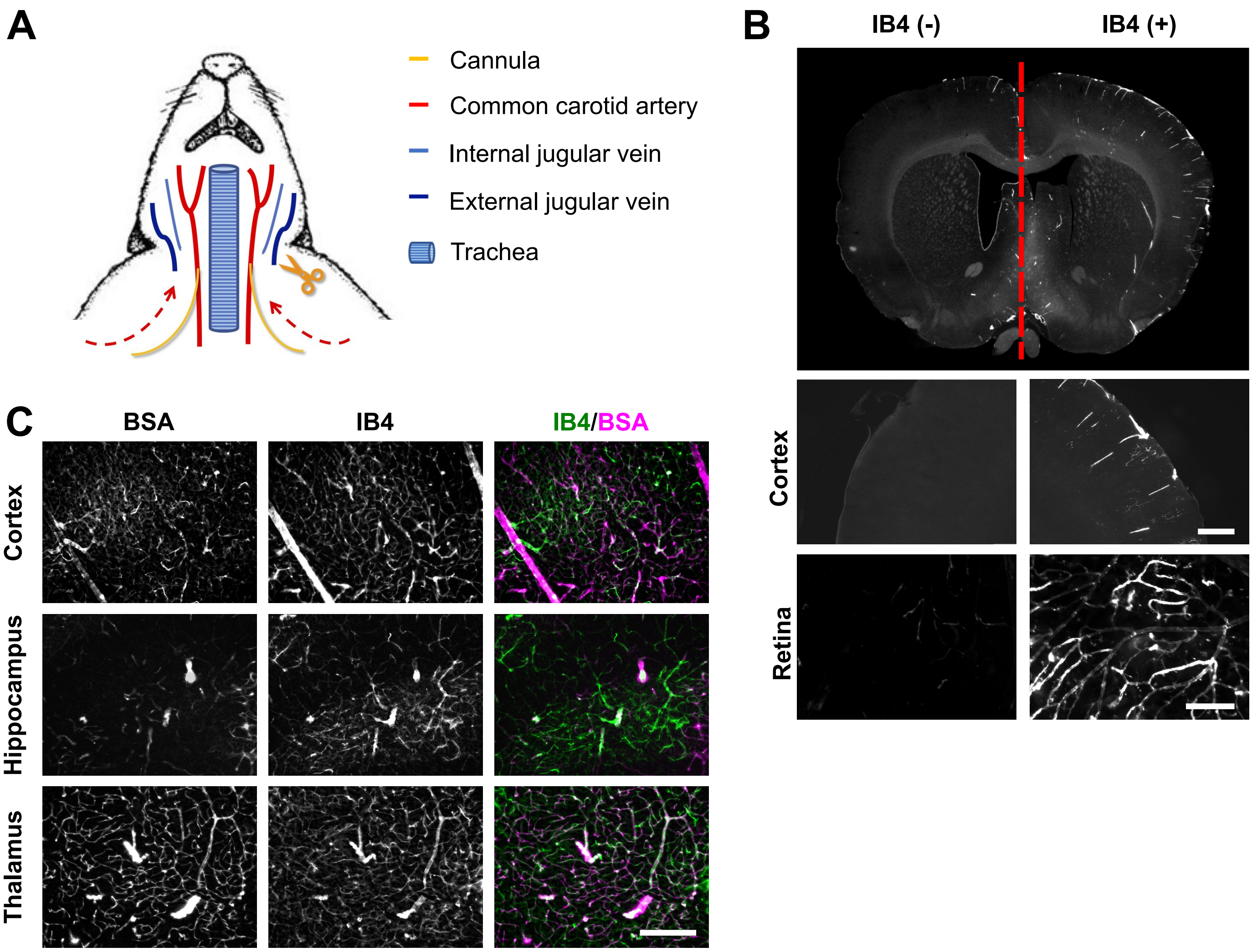

Figure S1. The dual carotid artery perfusion model. Related to Figure 1.
(**A**) Schematic of the experimental setup for rats and mice. Shortly after death, the two common carotid arteries were cannulated and the jugular veins sectioned. Heads were then perfused with saline containing heparin, followed by a cardioplegic solution, shown to preserve the vasculature and the BBB[18]. Subsequently, heads were incubated by perfusion with cardioplegic solution containing tracer molecules, METH, DOX, and AFL (or in control sides their respective vehicles).
(**B**) Both sides of a rat were perfused at equal pressure. Fluorescent IB4 was included in the perfusate of the right carotid artery. After 1 h, heads were perfused fixed, brains isolated and sections examined by epifluorescence microscopy. Note that IB4 only appeared in the right brain hemispheres and the right retinal vasculature, demonstrating that mixing of perfusates did not occur at the Circle of Willis. Shown are representative images of a single sectioned brain. Scale bars, 500 µm.
(**C**) Rat heads were perfused using the dual carotid artery model as described. EB-Alb was included with the perfusate and left within the heads for 1 h. Heads were perfused fixed, sectioned and the vasculature stained using IB4 before analysis by epifluorescent light microscopy. Note that EB-Alb was retained within the vasculature in all brain areas shown, indicating preservation of the BBB. Shown are representative images of 3 independent experiments. Scale bar, 50 µm.

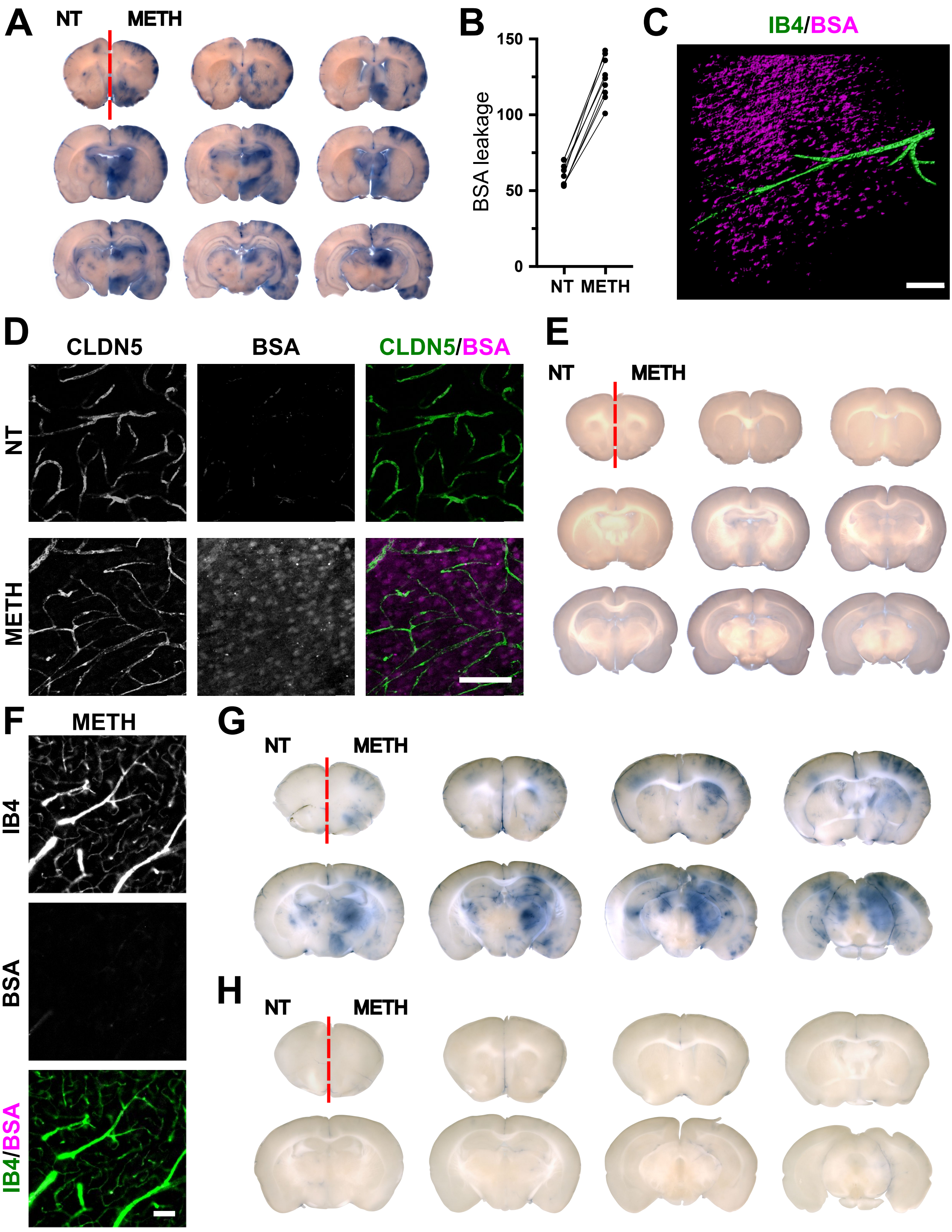

Figure S2. METH-induced leakage in the dual carotid artery perfused rats and mice. Related to Figure 1.
(**A, B**) As Figure 1 A, showing the full set of coronal sections (A) and the EB-Alb intensities of the METH- and non- treated hemispheres from each individual section (B). Shown is a section profile representative of n=5 independent experiments.
(**C**) As Fig 1B, showing a 3-D rendering of confocal stacks (optical thickness 60 μm) from a representative branching microvessel and demonstrating extravasation of EB-ALB into the parenchyma. Scale Bar, 10 μm.
(**D**) As in Figure 1 B but counterstained for CLDN5, indicating integrity of CLDN5 and extravasation of EB-ALB. Scale Bar, 100 μm.
(**E**) As Figure 1 C, showing the full set of coronal sections. Shown is a section profile representative of n=3 independent experiments.
(**F**) Brains were treated cold as described for Figure 1 C and coronal sections counterstained using IB4 (green). Shown are METH-treated fields from a representative thalamus. Scale bars, 100 μm.
(**G**) As Figure 1 G, showing a full set of representative coronal sections from WT mice. Shown is a section profile representative of n=3 independent experiments.
(**H**) As Figure 1 G, showing a full set of representative coronal sections from CAV1 -/- mice. Shown is a section profile representative of n=3 independent experiments.

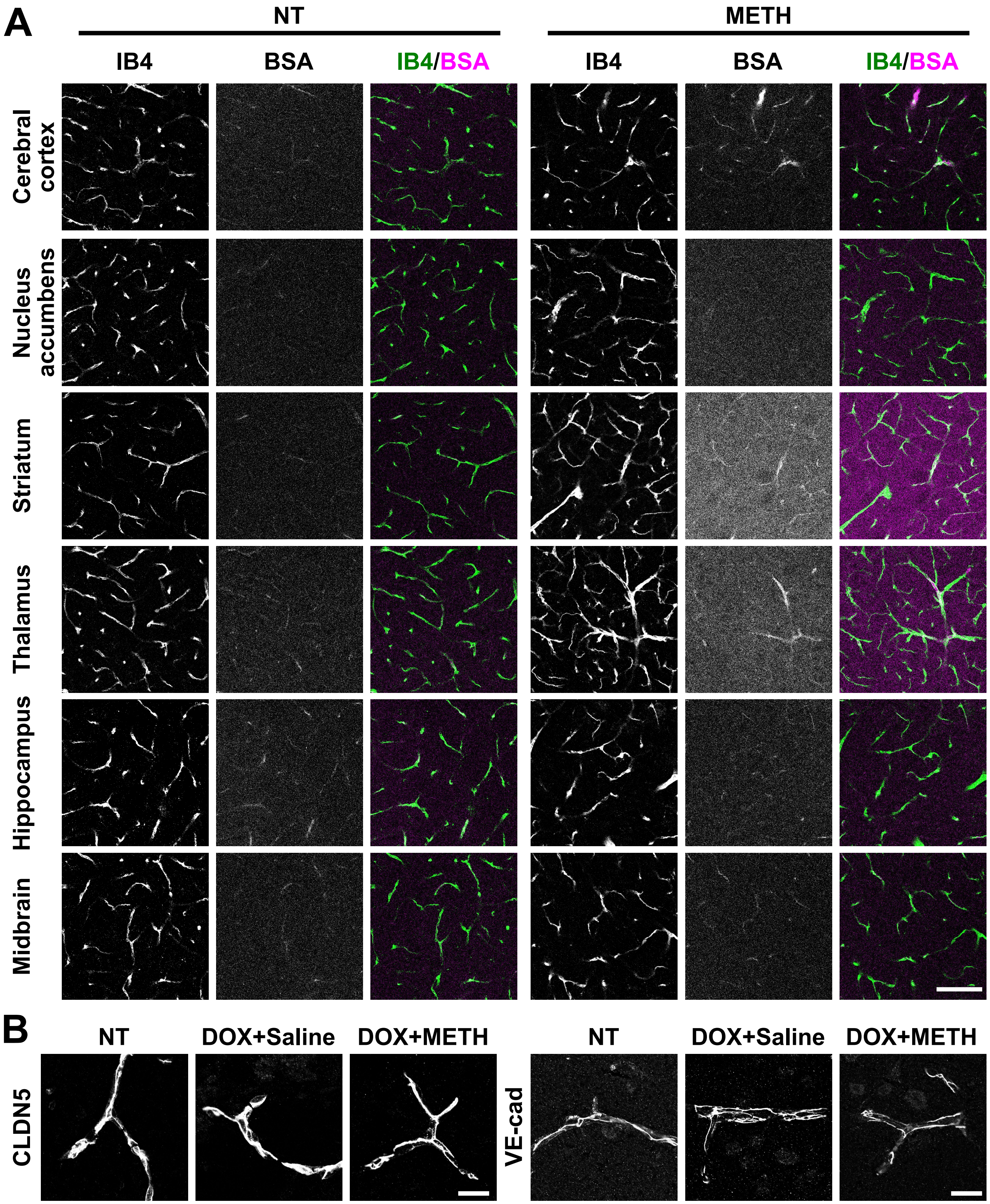

Figure S3. METH-induced BBB breakdown in mice. Related to Figure 2.
(**A**) As Figure 2C, D, showing representative images from the indicated brain regions and enhanced leakage of BSA-FITC in all brain areas analysed. Scale bars, 100 μm.
(**B**) CLDN5 and VE-cad staining of brain microvessels as Figure 2E, except that Cav1 -/- mice were used. Note that junctional staining is unaltered by METH. Scale bars, 20 μm. This is a set of representative images from n=3 independent experiments.

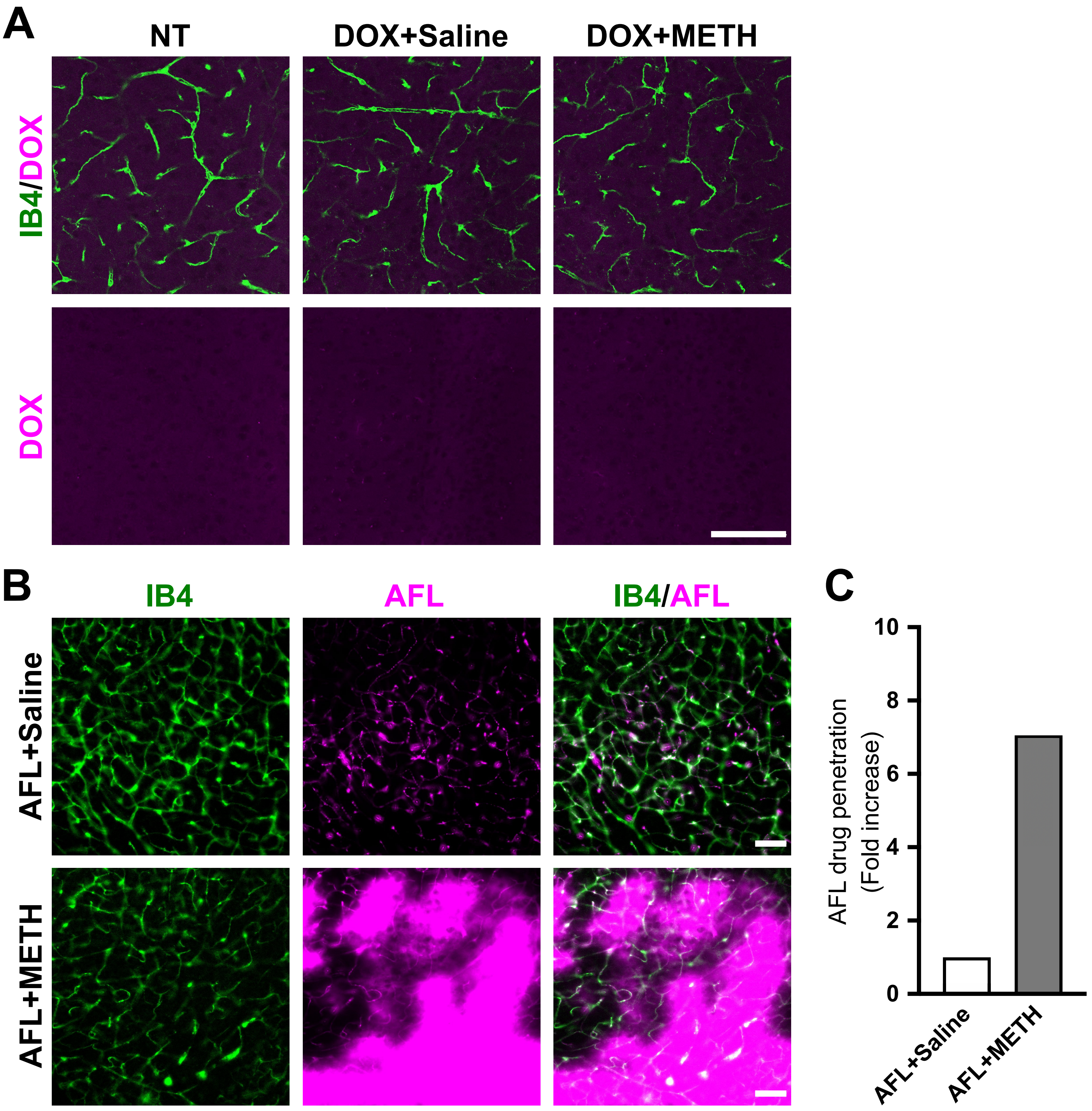

Figure S4. METH-induced drug transport across the BBB. Related to Figure 3.

(**A**) Cav1 -/- mouse brains were perfused *ex vivo* through both carotid arteries with cardioplegic solution containing DOX (10 µg/mL) and METH (1 µM) v saline in opposing sides as indicated. After 60 min the vasculature was flushed and brains perfused fixed, sectioned, stained, imaged and quantified. Shown is IB4 staining (green) of thalami representative of n=3 independent experiments. DOX was detected by virtue of its autofluorescence (magenta). Scale bars, 100 µm.

(**B, C**) A rat brain was perfused *ex vivo* through both carotid arteries with cardioplegic solution containing AFL (0.5 mg/mL) and METH (1 µM) vs. saline in opposing sides as indicated. After 60 min the vasculature was flushed and brains perfused fixed, sectioned, stained, imaged and quantified. Shown is the the IB4 (green) of the thalamus. AFL was revealed by staining using goat polyclonal anti-human IgG Fc antibody (B, magenta). Scale bars, 100 µm. (C) Quantification of fluorescent intensity in sections as shown in (B). Shown is mean AFL fluorescence normalised to vascular areas from 3 individual brain sections.

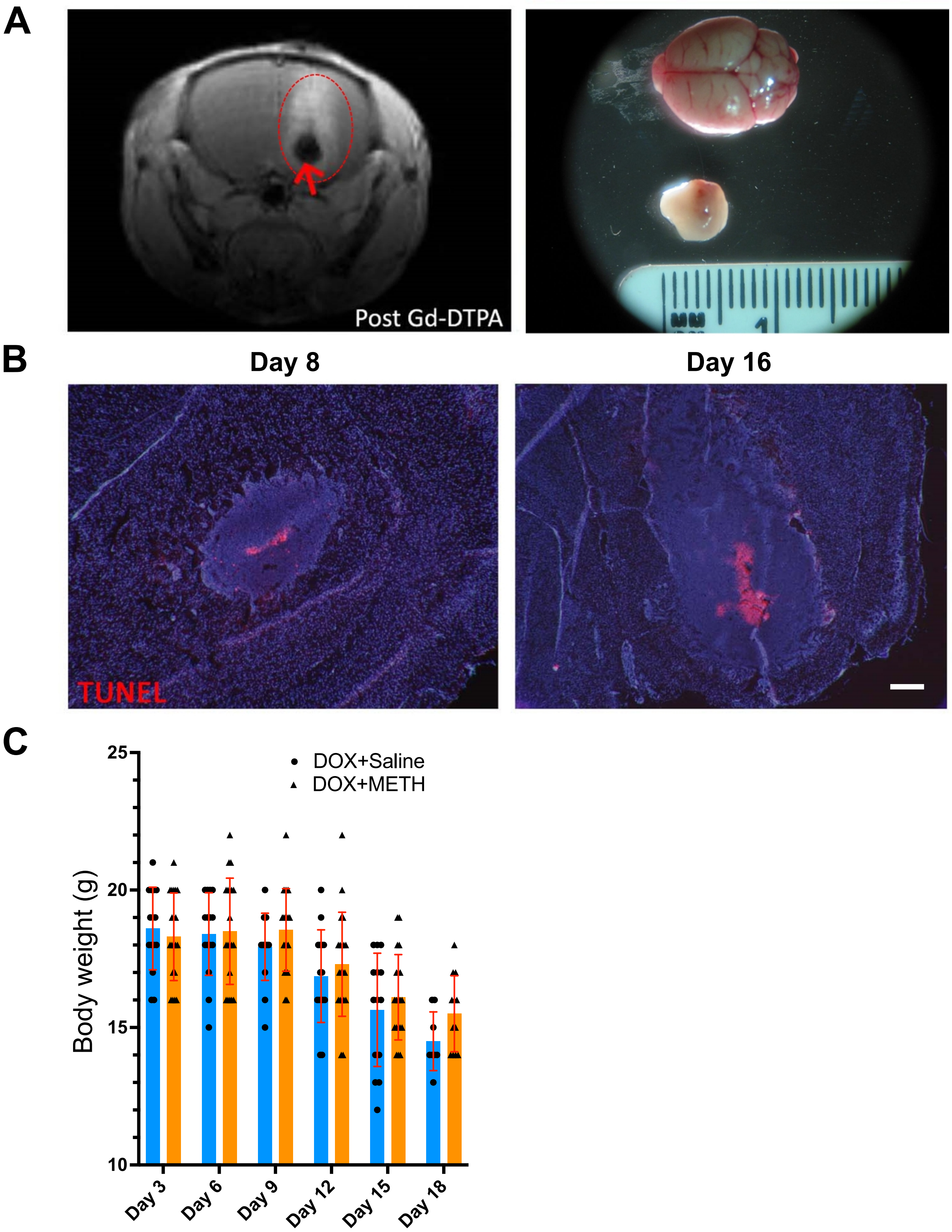

Figure S5. Orthotopical D270 glioblastoma in Balb/c mice. Related to Figure 3.
(**A**) Human D270 cells were injected into the right caudate-putamen region of an athymic nude mouse. On day 16 the mouse was subjected to T1-weighted MR images following Gd-DTPA (Gadolinium diethylene-triamine penta-acetic acid) administration. The arrow indicates the non-perfused necrotic core (left panel). The dotted line shows the approximate outline of the primary tumour, within which there is substantial leakage. The tumour was then resectioned and is shown next to a brain of an uninjected age-matched control animal (right panel).
(**B**) As in A except that brain sections containing tumours were analysed by TUNEL assay on day 8 and day 16. Note the increasing TUNEL positivity at the core of the tumour. Scale bars, 200 µm.
(**C**) Body weight over time of DOX+saline (blue) and DOX+METH (orange) mice described in Figure 3C shown as mean ± SD.

METH dose (i.p.)	Plasma concentration (µM)	
	30 min	60 min
**0.75 mg/kg**	n.d.	0.21±0.09
**2.5 mg/kg**	n.d.	0.75±0.11
**7.5 mg/kg**	5.26±0.69	2.94±0.87

Table ST1. METH plasma concentrations following i.p. dosing of mice *in vivo*. Related to Figure 2 and 3. C57BL6 mice were injected i.p with the indicated doses of METH. Plasma was isolated at the indicated times and METH content determined by LC-MS/MS (as described in the Method section). Data points are means ± SEM from 3 independent experiments.
